# Residual effect of commonly used fungicides in strawberries on *Amblyseius swirskii*, *Neoseiulus cucumeris*, and *Neoseiulus californicus* (Mesostigmata: Phytoseiidae)

**DOI:** 10.1007/s10493-024-00928-1

**Published:** 2024-06-13

**Authors:** Allan Busuulwa, Alexandra M. Revynthi, Oscar E. Liburd, Sriyanka Lahiri

**Affiliations:** 1 Entomology and Nematology Department, University of Florida, Gulf Coast Research and Education Center, Wimauma, FL USA; 2 Entomology and Nematology Department, University of Florida—Tropical Research and Education Center, Homestead, FL USA; 3 Entomology and Nematology Department, University of Florida, Gainesville, FL USA

**Keywords:** Predatory mites, *Fragaria x ananassa*, *Scirtothrips dorsalis*, Integrated pest management, Side effects, Biocontrol

## Abstract

Florida’s strawberry industry is currently valued at $511 million annually but faces challenges from pathogens and arthropod pests especially *Tetranychus urticae* Koch (twospotted spider mite) and *Scirtothrips dorsalis* Hood (chilli thrips). Predatory mites, particularly *Neoseiulus cucumeris* Oudemans, *Neoseiulus californicus* McGregor, and *Amblyseius swirskii* Athias-Henriot, play a crucial role in pest management. However, there are concerns regarding how these biological control agents are affected by fungicides used in current pathogen management strategies. This study assessed the residual effects of commonly used fungicides in strawberries on the survival, feeding, and oviposition of these predatory mites. Commercially sourced predatory mites were reared on *S. dorsalis* larvae, and gravid female predators placed on fungicide treated strawberry leaf discs in a Munger cell for 120 h. Fungicides tested included two formulations of Captan, hydrogen peroxide + peroxyacetic acid, cyprodinil + fludioxonil, tetramethylthiuram disulfide, cyflufenamid and a control. All fungicides tested had an impact on the survival, feeding, and oviposition of the predators. Among the fungicide treatments, the lowest predator survival was observed in the cyprodinil + fludioxonil treatment, while the highest was observed in the hydrogen peroxide + peroxyacetic acid and tetramethylthiuram disulfide treatments. In all treatments, feeding and oviposition greatly varied among predators; specifically, *N. cucumeris* and *A. swirskii* had the lowest prey consumption, while *N. californicus* had the highest. These findings highlight the potential incompatibility between fungicides and predatory mites and demonstrate the need for the development of a fungicide rotation program tailored to the different susceptibilities of predators to fungicides.

## Introduction

Strawberry (*Fragaria* x *ananassa* (Rosaceae) production is one of the major economic activities in Florida, with annual revenue of approximately $511 million as of 2023 (USDA/NASS-2023). Florida has been ranked as the largest producer of winter strawberries and the second largest producer of strawberries in the USA(Guan et al. 2016; Huang et al. 2022). Nonetheless, this industry faces major challenges from both arthropod pests and pathogens. In Florida, the strawberry arthropod pest complex is comprised of several pests including various thrips species such as *Frankliniella occidentalis* Pergande, *Frankliniella bispinosa* Morgan, and the invasive *Scirtothrips dorsalis* Hood (Thysanoptera: Thripidae), with the latter being the prominent pest (Lahiri and Panthi 2020; Panthi and Renkema 2020; Panthi et al. 2021). Additionally, the pest complex includes various phytophagous mite species with *Tetranychus urticae* Koch (Trombidiformes: Tetranychidae) (twospotted spider mite) being the predominant mite pest (Liu et al. 2016; Akyazi and Liburd 2019; Gireesh et al. 2022; Lahiri et al. 2022; Montemayor et al. 2023).

Adding to the already existing arthropod challenges affecting the strawberry industry in Florida, disease causing pathogens are another important factor to consider. Some of the most important diseases of strawberries in Florida include anthracnose fruit rot (AFR) caused by various species of *Colletotrichum* (Rebello et al. 2022), strawberry powdery mildew caused by *Podosphaera aphanis* (Wallr.) U. Braun and S. Takam (Erysiphaceae) (Onofre et al. 2021), angular leaf spot caused by *Xanthomonas fragariae* (Xanthomonadaceae) (Roach et al. 2016), and most recently reported *Neopestalotiopsis* spp. causing strawberry fruit rot.(Baggio et al. 2021a; Kaur et al. 2022).

Strawberry growers in Florida use a wide range of disease management strategies that integrate innovative technologies such as thermotherapy of strawberry transplants (Baggio et al., 2021b) and UV-C technology (Onofre et al. 2022; Mello et al. 2022; Montemayor et al. 2023) with planting disease resistant strawberry varieties (Kennedy et al. 2013; Roach et al. 2016; Whitaker et al. 2017). However, the use of fungicides remains crucial for effective disease control in strawberries (Oliveira et al. 2020; Marin et al. 2021). Some commonly used fungicides in strawberry production include: Captan, used to manage botrytis fruit rot (Legard et al. 2001), Captan Gold, used against Anthracnose (*Colletotrichum acetatum*) and botrytis (Gama et al. 2023), Oxidate (hydrogen peroxide + peroxyacetic acid), used to manage alternaria, angular leaf spot, crown rot, leaf blight, fruit rot, and powdery mildew (Peres et al. 2023); Switch (cyprodinil + fludioxonil), commonly applied at planting to control crown and root rot and anthracnose (Acosta-González et al. 2023); Thiram (tetramethylthiuram disulfide), used specifically against gray mold (*Botrytis cinerea*) (Peres et al. 2023) and Torino (cyflufenamid), used against powdery mildew (Peres et al. 2023).

To manage arthropod pests especially *F. occidentalis*, *S. dorsalis* and *T. urticae*, many strawberry growers rely on a combination of insecticides and phytoseiid mites. The predatory mites commonly used include *Amblyseius swirskii* (Athias-Henriot), *Neoseiulus cucumeris* Oudemans and *Neoseiulus californicus* McGregor (Mesostigmata: Phytoseiidae). *Amblyseius swirskii*, *N. cucumeris*, and *N. californicus* are generalist predators (McMurtry et al. 2013) capable of feeding on various mite species (Rhodes and Liburd 2006), thrips (Arthurs et al. 2009), and pollen (Delisle et al. 2015). Their ability to suppress agriculturally important pests has facilitated their commercialization, making them some of the most widely used biological control agents in both fields and greenhouses (Lahiri and Yambisa 2021) Additionally, their capability to survive on pollen enables them to maintain stable populations even when pest populations fluctuate, therefore providing consistent pest suppression. These combined attributes have led to their dominant use for *S. dorsalis* suppression in strawberries.

In a typical strawberry season, growers apply fungicides on a weekly basis and introduce predators twice during the season. However, operational approaches greatly vary depending on the specific operation and pest density. This is done so as to efficiently manage both insect and pathogen pests. Similar to many synthetic compounds like insecticides and herbicides, fungicides exhibit various side effects that may negatively impact the fitness of the released predatory mites (Reiff et al. 2021). For instance, combinations of ametoctradin + dimethomorph and propamocarb + dimethomorph fungicides were found to be highly toxic to deutonymphs of *A. swirskii*, causing mortalities of 77.5% and 60.00% respectively (Ersin et al. 2020). Luna® Tranquility (fluopyram + pyrimethanil) a polyfunctional fungicide was reported to cause 90.6–100% mortality of *A. swirskii* and *N. cucumeris*, respectively, 10 h after treatment, the same study reported moderate toxicity to *Phytoseiulus persimilis* Athias-Henriot (Sukhoruchenko et al. 2021). The dithiocarbamate mancozeb, a fungicide commonly used in many vineyards was found to be moderately toxic to the predatory mite *Typhlodromus pyri* Scheuten (Auger et al. 2004). In bees, some fungicides have been reported to cause disruptions in bee nest recognition (Artz and Pitts-Singer 2015). However, not all fungicides, are harmful, for example, field concentrations of copper fungicides i.e., copper hydroxide, copper gluconate and 30% DT (copper succinate + copper glutarate + copper adipate) were found to have low to moderate toxicity to *N. cucumeris* (Mao et al. 2011). The fungicides metiram + pyraclostrobin, azoxystrobin, copper hydroxide, and mancozeb were found to have low toxicity to *N. californicus* (Silva et al. 2019). Put et al. 2016; found that fungicides such as fluopyram + tebuconazole, boscalid, kresoxim-methyl, cyprodinil + fludioxonil, and dodemorph were harmless to the predatory mite *Euseius gallicus* Kreiter and Tixier (Acari: Mesostigmata).

In light of this information, it is therefore crucial to investigate the compatibility of fungicides used for disease management in strawberries with the predatory mites used for *S. dorsalis* suppression in the same crop. Understanding this interaction is important because it can guide the development of effective fungicide rotation programs that minimize the side effects of fungicide on predatory mite populations. Therefore, the main objective of this study was to determine the compatibility of fungicides with predatory mites by comparing their impact on the survival, feeding, and oviposition of *A. swirskii*, *N. cucumeris*, and *N. californicus*. We hypothesized that commonly used fungicides in strawberry would negatively impact the predatory mites leading to a reduction in their feeding, oviposition, and survival.

## Materials and methods

### Strawberry plant rearing

Strawberry plants (*Fragaria x ananassa*, Rosaceae) used in the experiment were grown in plastic pots, each with dimensions of 14.6 cm x 15.24 cm (Kord Regal Standard plastic pots, The HC Companies, Twinsburg, OH, USA). The pots were filled with general-purpose growing medium (ProMix BX, Sun Gro Horticulture, Agawam, MA, USA) and arranged on trays measuring 30.48 cm x 40.64 cm (Choice Teal Plastic Fast Food products, Michigan, USA), with each tray accommodating four plastic pots. These trays were subsequently placed inside a rearing and observation cage, which measured 61 cm x 61 cm x 61 cm (Bioquip, Compton, CA, USA). The entire setup was put in a growth chamber with a controlled environment, maintained at a constant temperature of 25 ± 1 °C, relative humidity (RH) of 65 ± 5%, and a 14:10-hour light-to-dark photoperiod. The strawberry plants were watered twice a week by adding approximately 1.5 L of water mixed with 16 ml of fertilizer (J R Peters Classic 20-20-20 N-P-K All Purpose Fertilizer, Allentown, PA, USA) to the trays. The plants were allowed to grow for a duration of 6 weeks before being used in the experiments.

### Predatory mite rearing

Predatory mites used in this experiment included *A. swirskii*, *N. cucumeris*, and *N. californicus*. Stock cultures of the predatory mites were obtained from ARBICO Organics (Tucson, AZ). On arrival to the laboratory, they were kept separately in their original containers in a 45 L ice cooler (Island Breeze Family cooler, Igloo, Katy Texas) packed with 8 Foam refrigerants (Polar Tech Industries INC, Genoa, IL USA) that had been kept in a freezer overnight.

To establish the laboratory colonies used in the bioassays, two hundred gravid females of each mite species were transferred onto separate rearing arenas using a 3/0 fine paint brush (Artist Brush Keep Smiling, Shenzhen Eseng International Co., LTD., Shenzhen, China). Female predators were identified as gravid based on their distinctively enlarged and round-shaped opisthosomas. The rearing arenas were modelled closely to those described by Helle and Sabelis (1985). The arenas consisted of plastic dish pans measuring 35.6 × 29 × 12 cm (Greenbrier International. Inc. USA), half filled with distilled water. QEP extra-large multipurpose sponges measuring 19 × 14 × 2.5 cm (Boca Raton, FL, USA) were then placed in each dish pan and allowed to absorb water. Black polystyrene flexible plastic board sheet (MEGA Format, Brooklyn, NY, USA) measuring 12 × 8 cm was placed on top of the sponges to provide a surface that predatory mites would inhabit. To prevent mites from escaping, the borders of the plastic board sheets were covered with moist nonsterile cotton (Fisher Scientific, NJ, USA).

To provide a suitable environment for oviposition, some cotton fibers were adhered to small plastic sheets, creating triangular structures under which mites could oviposit. The arenas were transferred into a growth chamber and kept at 25 ± 1 °C, 70 ± 5% RH, and a photoperiod of 14:10 (L: D) h. The established colonies were feed with first and second instar larvae of *S. dorsalis* by transferring approximately 300–400 larvae onto the arena every 48 h using a paint brush. *Scirtothrips dorsalis* used as a food source for the mites was obtained from the laboratory colony maintained on cotton plants in a growth room at 25 ± 1 °C, relative humidity (RH) of 65 ± 5%, and 14:10 h L: D.

To obtain mites of the same age, 60 pairs of gravid female predatory mites were randomly selected from the established colony and transferred onto separate rearing arenas and allowed to oviposit for 24 h. After this period, the females were removed from the arena and the new arenas kept in a growth chamber at 26 ± 1 °C, 70 ± 5% RH, at a photoperiod of 14:10 (L: D) h to facilitate hatching of the eggs. After hatching, the predatory mite nymphs were feed with first and second instar larvae of *S. dorsalis* by brushing approximately 200 larvae onto the rearing arena. This process was repeated every 48 h for a total of 8 days, continuing until the predatory mites completed their development into adults and initiated oviposition.

### Fungicides

Six fungicides commonly used in management of key diseases in strawberries in Florida were selected for testing (Table 1). These included hydrogen peroxide + peroxyacetic acid (OxiDate 5.0, contact fungicide, BioSafe Systems, East Hartford, CT, USA), cyprodinil + fludioxonil (Switch 62.5 WG, systemic fungicide, Syngenta, Greensboro, NC, USA), tetramethylthiuram disulfide (Thiram Granuflo®, broad-spectrum fungicide, Lanxess, Cologne, Germany), cyflufenamid (Torino®, Gowan, Yuma, AZ, USA) and two formulations of N-trichloromethylthio-cyclohexene-1,2-dicarboximide (Captan), Captan 50 W (Drexel Chemical Company, Memphis, TN, USA) and Captan Gold® 4 L (Adama, Raleigh, NC, USA). The concentration of the active ingredient in Captan Gold® 4 L is slightly lower than of Captan 50 W, with percentages of 38.75% and 48.9%, respectively. For simplicity, we refer to Captan 50 W as Captan and Captan Gold® 4 L as Captan®.

**Table 1:** Fungicides used in the study, including their trade names, active ingredients, active ingredient composition, FRAC code and application rate used

Fungicide Trade Names	Active Ingredient (Ai)	Ai Composition	Group Name	FRAC Code	Application Rate
Captan 50 W	N-trichtoromethylthio ~ cyclohexene-1,2-dicarboximide (Captan)	48.90%	Phthalimides	M 4	6.7 kg/ha
Captan Gold® 4 L		38.75%			7.02 L/ha
OxiDate® 5.0	Hydrogen Peroxide Peroxyacetic Acid	27.11% 2.0%	Not Specified	Not Specified	1.4 L/ha
Switch 62.5WG	Cyprodinil Fludioxonil	37.5% 25.0%	Anilino Pyrimidines and Phenylpyrroles	9 + 12	0.98 kg/ha
Thiram Granuflo®	Tetramethylthiuram disulfide	75.0%	Dithiocarbamate and relatives	M3	4.93 kg/ha
Torino®	Cyflufenamid	10%	Phenyl acetamide	U6	0.25 L/ha

FRAC- Fungicide Action Committee. FRAC Codes: M- Multisite Activity, U- Unknown mode of action

### Bioassays for residual contact toxicity of fungicides to predatory mite adult females

Strawberry leaves used in the experiment were those obtained from *S. dorsalis* free plants from growth the chamber. Leaf discs, measuring 12 mm in diameter, were cut from these strawberry leaves and subsequently, dipped for a duration of 10 s in a fungicide solution, which had been prepared based on the manufacturer’s maximum recommended application rate for strawberries (Table 1). The control treatment comprised of strawberry leaf disc treated with distilled water. Following the immersion, the treated leaf discs were allowed to air dry for 1.5 h before being introduced into the arena.

Munger cells used in the experiment were similar to those described by Helle and Sabelis 1985; and Argolo et al. 2020. The arenas consisted of two transparent acrylic glass plates measuring 75 mm by 26 mm. One piece of the glass plates had a circular hole measuring 12.7 mm in diameter made in the center in such a configuration that the hole would fit within the outline of the leaf disc used in the essay. The other glass plate of the same size was used to form the base of the construction. On this plate, a layer of moist cotton was laid on which a leaf disc with the abaxial surface facing downwards was placed. The glass plate with holes was subsequently placed carefully on the leaf disc forming a stack sandwich. In order to maintain constant contact between the predatory mites and the fungicide, the glass plate with a central hole was also subjected to the fungicide treatment. The plate was dipped into the solution for 10 s and subsequently left to air dry before being utilized in the arena.

A non-starved 8-day-old female predator from the colony was individually introduced into each arena by placing it directly onto the treated strawberry leaf disc. Ten first and second *S. dorsalis* instar larvae were subsequently placed inside the arena with the predatory mite to act as food source. A microscopic glass slide (Fisher Scientific, USA) was placed on top of the stack to prevent mites from escaping and to allow for easy observation under a stereomicroscope. Subsequently, the whole stacked was secured with binder clips and the setup placed in a growth chamber at 26 ± 1 °C, 70 ± 5% RH, and a photoperiod of 14:10 (L: D) h.

Every 24 h, *S. dorsalis* larvae were added to each arena to replace the consumed prey. The number or predatory mites alive, *S. dorsalis* larvae consumed, and number of eggs laid were recorded at 24, 48, 72, 96 and 120 h. *Scirtothrips dorsalis* larvae preyed upon by the predator displayed distinct characteristics, including a shriveled and deflated appearance, often accompanied by desiccation. These characteristics allowed easy differentiation from larvae that died naturally. Each treatment consisted of 10 repetitions, and the entire experimental setup was repeated twice. (two trials performed).

### Data Analysis

Generalized Linear Mixed Effects Models (GLMM) were used to examine the effects of residual toxicity of commonly used fungicides in strawberry production on the survival, feeding and oviposition of *A. swirskii*, *N. cucumeris*, and *N. californicus*. All models were fit using the glmmTMB package (Brooks et al. 2017) in R- version 4.3.0 (R Core Team 2023). Upon model selection, analysis of deviance was performed using the Anova function from the car package (Fox et al. 2013). In addition, when we detected a significant interaction term, we proceeded to perform linear contrasts using the Tukey adjustment (Tukey’s HSD test: *p* < 0.05) for multiple comparisons using the emmeans function from the emmeans package (Lenth 2020).

### Survival

To determine the effects of residual toxicity on survival of predatory mites, we fit six models. Model 1 (Mod1) included survival as a response variable, fungicide treatment, duration (hours), species of predatory mite (spp) and trial as fixed effects with repetition as a random effect. The trial was treated as a fixed effect due to model convergence issues when nested within the repetition (block). In addition, the model did not include interactions, and the binomial distribution was used to model the underlying error distribution. Subsequently, we proceeded to fit the second model (Mod2) that was similar to Mod1 but included a quadratic term (duration^2^) to capture the curvature in survival trend over time.

We continued to fit another model (Mod2_1) that was similar to model Mod2 but allowed for each treatment to exhibit its own curvature. Additionally, we fit model 3 (Mod3) that was similar to Mod2 but included interactions for all fixed effects and a quadratic term. Given that the model included time (duration of exposure) as a fixed effect, it was inspected for autocorrelation using the Check_autocorrelation function from the performance package (Lüdecke et al. 2021), residuals were found to be autocorrelated (*p* < 0.001).

To address the autocorrelation of residuals in the model, we fit model 4 (Mod4) that included species of predatory mites, fungicide treatment, duration (hours), their interactions, a quadratic term and trial were also included as fixed effects with an underlying First-order Autoregressive (AR1) covariance structure among measures taken from the same arena over time. Finally, we fit model 4_1 (Mod4_1) that was similar to Mod4 but only included species: treatment interaction and allowed for each treatment to have its own curvature. The treatment: duration and the three-way interaction; species: treatment: duration were removed because they were found to be non-significant in Mod4 (χ^2^ = 8.62, df = 6, *P* = 0.196 and χ^2^ = 7.23, df = 12, *P* = 0.84 respectively).

To choose the best model, we performed model selection using AIC i.e., Akaike Information Criterion(Cavanaugh and Neath 2019) via the AICc function found in the MuMIn package (Kamil Barton 2023). We proceeded with model 4_1 (Mod4_1) because it had the lowest AIC value (Table 2) after which analysis of deviance was performed. To determine model fitness, we further visually assessed model residuals using the DHARMa package (Florian Hartig 2022). We found that the assumptions of normality and homoscedasticity had been reasonably met.

**Table 2:** AIC values corresponding to survival models built

Model Name	Degrees of Freedom (df)	AIC Value
Mod1	12	1275.648
Mod2	14	1263.260
Mod2_1	20	1267.963
Mod3	44	1278.296
Mod4	46	1266.945
Mod4_1	32	1254.007

To determined predatory mite survival, a post hoc means contrast was performed, comparing probability of predatory mite survival under different treatments. To further investigate the effect of duration (time) of exposure to fungicides on the probability of predatory mite survival, a post hoc means contrast was done comparing survival of different predatory mite species at all time intervals. All the aforementioned contrasts were formed by aggregating data across all other factors in the model. Additionally, the means and confidence intervals (CI) were back transformed before being presented in both textual descriptions and figures.

### Feeding

Proportion of prey consumed was calculated as total number of prey consumed divided by total number of prey provided. Using a similar approach in modeling survival, we fit two models. Model 1 (md_1) included treatment, species of predatory mites, trial and duration as fixed effects with repetition set as a random variable. Given that the response variable was in form of proportions, the underlaying error distribution was modeled using the Ordered Beta Regression distribution (Kubinec 2022). Model 2 (md_2) included all fixed effects in model 1 with their respective interactions. In addition, we added a quadratic term to capture the curvature in feeding trend over time. Fitting a model that allowed each treatment to possess its own curvature resulted into model convergence. Subsequently, model selection was done using AICc function. Model 2 was selected because it had a lower AIC value compared to Model 1 (md_1, df = 18, AICc = 385.09, md_2; df = 48, AICc = 260.11).

Additionally, model fit was evaluated by visually inspecting the residuals. We found that the assumptions of normality and homoscedasticity were reasonably satisfied. An analysis of deviance was subsequently performed followed by linear contrasts to estimate the effect of fungicides and duration of exposure on the oviposition of predatory mites.

### Oviposition

To evaluate daily oviposition, we fit a model using the number of eggs produced as the dependent variable. The model accounted for various factors, including the fungicide treatment, species of predatory mite, and their interaction and trial. Furthermore, the model allowed each treatment to exhibit a unique temporal trend. Random effects were incorporated for repetition, and an AR1 covariance structure was applied to address autocorrelation. The negative binomial distribution was used to accurately represent the underlying error distribution within this model.

Furthermore, we assessed model fit by visually examining the residuals, which indicated reasonable adherence to the assumptions of normality and homoscedasticity. We proceeded to check for overdispersion using the check_overdispersion function from the performance package. The model was not overly dispersed (dispersion ratio = 0.886, Pearson’s χ^2^ = 905.335, *p* = 0.996). An analysis of deviance was subsequently conducted, followed by linear contrasts to estimate the impact of fungicides and exposure duration on the oviposition of predatory mites.

## Results

### Survival

Following the chi-square (χ2) test that was conducted to determine the effect of predator species, fungicide treatments, and duration of exposure to fungicides on the survival of predatory mites. The results showed significant effects of predator species (χ2 = 6.89, df = 2, *p* = 0.032), fungicide treatments (χ2 = 56.85, df = 6, *p* < 0.001), and the duration of exposure to fungicides (χ2 = 38.07, df = 1, *p* < 0.001) on the survival of predators. Additionally, significant interaction effects were observed between predatory mite species and fungicide treatments (χ2 = 34.46, df = 12, *p* < 0.001). Secondly, there were no differences in survival estimates between the two experimental trials carried out (χ2 = 1.72, df = 1, *p* = 0.19), therefore suggesting that survival estimates were similar across each trial, an indication of the repeatability of the experiment. The significance of the predatory mite species-fungicides interaction revealed that the survival of each species was uniquely influenced by specific treatment applications, implying that predator species did not respond uniformly to the same treatments.

**Fig. 1:**
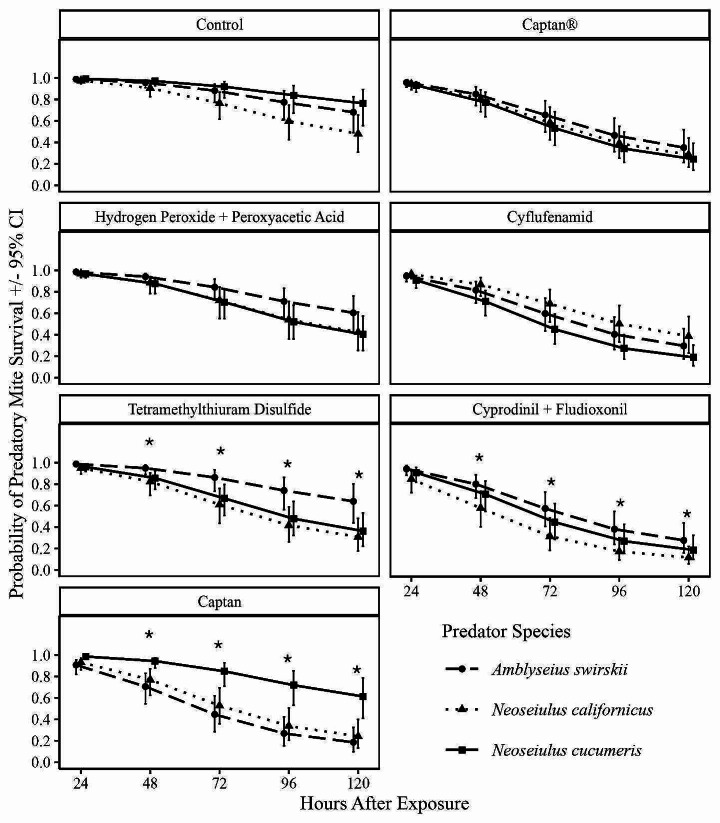
Survival of *A. swirskii*, *N. californicus*, and *N. cucumeris* exposed to different fungicide treatments over 120 h. Asterisk (*) indicates significant differences in survival among predator species at each individual time point (Tukey’s HSD test: *p* < 0.05)

The average pooled survival of all predatory mites was lowest in the cyprodinil + fludioxonil fungicide treatment (0.165, Confidence Interval [CI] 0.08–0.312) compared to the control treatment (0.741, CI 0.584–0.850). Among the fungicide treatments, the highest average survival for all predators was observed in treatments with tetramethylthiuram disulfide and hydrogen peroxide + peroxyacetic acid, while the overall average survival was similar for cyflufenamid, captan, and captan® treatments (Table 3). Considering the significant interaction between predatory mite and fungicide treatments, a comparative analysis of the three predatory mite species in identical fungicide treatments at each observational interval (every 24 h) showed varied results. *Amblyseius swirskii* had higher survival in the cyprodinil + fludioxonil and tetramethylthiuram disulfide treatments compared to *N. californicus* and *N*. *cucumeris* (Fig. 1). *Neoseiulus cucumeris* had higher survival in the captan treatment compared to both *A. swirskii* and *N. californicus*. However, there were no differences in the survival of the three predatory mites when exposed to captan®, cyflufenamid and hydrogen peroxide + peroxyacetic acid. Although the highest survival was observed in the control treatment, there was a general decline in survival of the predatory mites following prolonged exposed to the fungicide treatments (Fig. 1).

**Table 3:** Survival of predatory mite under different fungicide treatments across species and time

Fungicide Treatment	Survival Estimate	LCL	UCL
Control	0.741 a	0.584	0.850
Tetramethylthiuram disulfide	0.481 ab	0.321	0.646
Hydrogen peroxide + Peroxyacetic acid	0.456 abc	0.307	0.614
Cyflufenamid	0.320 bc	0.197	0.476
Captan	0.254 bc	0.132	0.432
Captan®	0.216 bc	0.120	0.362
Cyprodinil + Fludioxonil	0.165 c	0.08	0.312

*Note* The “Fungicide Treatment” column lists fungicides tested. “LCL” and “UCL” represent lower and upper credible limit intervals (Tukey’s HSD test: *p* < 0.05). Results are averaged across species and time. Intervals were back transformed from the logit scale. Tests were performed on the log odds ratio scale. Different letters denote differing estimates

### Feeding

The chi-square test performed revealed that exposure of predatory mites to fungicides significantly affected their consumption of *S. dorsalis* larvae (χ2 = 53.7086, df = 6, *p* < 0.001). Furthermore, notable differences were observed in the trend of *S. dorsalis* consumption by the predatory mites (χ2 = 80.8088, df = 2, *p* < 0.001). Additionally, the duration of exposure to fungicides had a significant impact on *S. dorsalis* larvae consumption by the three predators (χ2 = 772.6759, df = 1, *p* < 0.001), therefore indicating that each predator consumed different quantities of *S. dorsalis* larvae. The quadratic term incorporated into the model revealed a nonlinear trend in *S. dorsalis* consumption over time (χ2 = 2192.2278, df = 1, *p* < 0.001). However, the interaction among fungicides, predatory mites, and the duration of exposure was not significance (χ2 = 20.3812, df = 12, *p* = 0.06021).

**Fig. 2:**
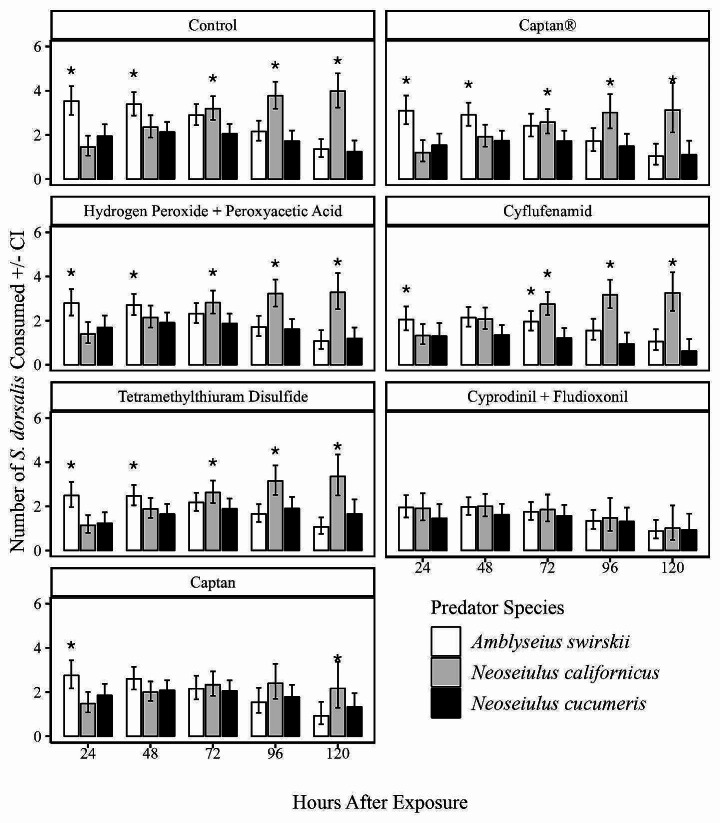
Number of *S. dorsalis* consumed by *A. swirskii*, *N. californicus* and *N. cucumeris* exposed to different fungicide treatments over time. Asterisk (*) denotes significant differences in prey consumption between the species at each individual time point (Tukey’s HSD test: *p* < 0.05)

The control treatment had the highest number of *S. dorsalis* larvae consumed (Fig. 2). Notably, in this treatment during the first 48 h of observation, *A. swirskii* had the highest number of *S. dorsalis* larvae consumed (3.5 CI: 3.04–4.05), compared to *N. californicus* (1.5 CI: 1.11–1.87) and *N. cucumeris* (1.9 CI: 1.5–2.4). Beyond this time point, the number of *S. dorsalis* consumed by *N. californicus* consistently increased while that of *N. cucumeris* and *A. swirskii* decreased (Fig. 2). A similar trend was observed when predators were exposed to hydrogen peroxide + peroxyacetic acid, tetramethylthiuram disulfide, captan® and cyflufenamid fungicide treatments. When exposed to captan, predatory mites exhibited a slightly different trend in *S. dorsalis* larvae consumption, In the first 24 h, *A. swirskii* consumed the highest number of *S. dorsalis* (2.1 CI: 2.2–3.3) compared to *N. californicus* (1.5 CI: 1.1–1.9) and *N. cucumeris* (1.8 CI: 1.5–2.3). However, at 48, 72, and 96 h, all three predators consumed similar proportions of prey. By the 120-hour mark, *N. californicus* had the highest number of prey consumed (2.1 CI: 1.3–3.3) compared to *N. cucumeris* (1.3 CI: 0.9–1.9) and *A. swirskii* (0.9 CI: 0.6–1.5). Conversely, the cyprodinil + fludioxonil treatment had the lowest number of *S. dorsalis* consumed among all the treatments, with all three predatory mites consuming relatively low numbers of *S. dorsalis* throughout the observation period (Fig. 2).

### Oviposition

**Fig. 3:**
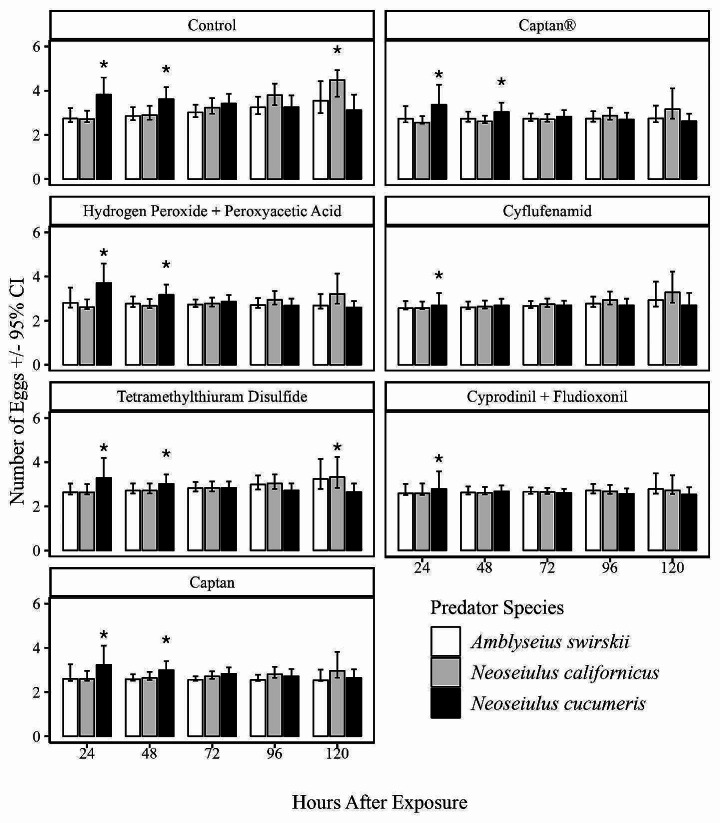
Number of eggs produced by *A. swirskii*, *N. californicus* and *N. cucumeris* exposed to different fungicide treatments over 120 h. The asterisk (*) indicates significant differences in prey consumption between predators at each individual time point (Tukey’s HSD test: *p* < 0.05)

Results from the chi-square test showed that exposure to fungicides significantly affected the oviposition of the three predatory mite species (χ2 = 11.1467, df = 2, *p* < 0.01). Additionally, there was a significant interaction between the fungicide treatments and the duration of exposure, demonstrating substantial variability in oviposition among the three predators over time (χ² = 66.9020, df = 7, *p* < 0.001). Among all the treatments, the highest oviposition was observed in the control treatment (Fig. 3), in this treatment, *N. cucumeris* had a significantly higher number of eggs produced (3.58 CI: 3.17–4.12) compared to *N. californicus* (2.65 CI: 2.58–2.80) and *A. swirskii* (2.72 CI: 2.61–2.91). However, the number of eggs produced by *N. cucumeris* gradually decreased with time while that of *N. californicus* and *A*. *swirskii* gradually increased. By 120 h, *N. californicus* had higher number of eggs produced compared to *A*. *swirskii* and *N. cucumeris* (Fig. 3). In the captan®, hydrogen peroxide + peroxyacetic acid and captan treatments, *N. cucumeris* had the highest number of eggs in the initial 48 h of observation, however beyond this point there were no differences in oviposition among the three predators. A similar trend was observed in the tetramethylthiuram disulfide treatment only that at 120 h of observation, *N. californicus* had the highest oviposition among the three predators. Cyprodinil + fludioxonil treatment relatively had the lowest number of eggs produced among all the fungicide treatments. In this treatment at 24 h of observation, *N. cucumeris* had the highest number of eggs, however beyond this point, similar number of eggs were observed among the predators.

## Discussion

Although applying fungicides is crucial for disease management in strawberries, it’s also vital to consider the potential side effects that they may have on predatory mites that released for *S. dorsalis* suppression in the same crop. Our findings indicate that exposure of *N. cucumeris*,*N. californicus* and *A. swirskii* to field level concentrations of cyprodinil + fludioxonil resulted in a significant reduction in their survival with less than 20% of the predators surviving after 120 h of exposure. Among all the tested fungicides, hydrogen peroxide + peroxyacetic acid had the least impact on the survival of the three predators. Most importantly, our findings indicated that survival of the predators after exposure to fungicides was different for each predatory mite species. For example, *A. swirskii* had better survival in the tetramethylthiuram disulfide treatment, compared to *N. cucumeris* and *N. californicus* while *N. cucumeris* had better survival in the captan treatment compared to. *A. swirskii* and *N. californicus* Among the three predators, *N. californicus* had the lowest survival rates in all the fungicide treatments.

While fungicides are not targeted at arthropods, their application can inadvertently affect many non-target organisms such as bees and predatory mites (Ioriatti et al. 1992; Artz and Pitts-Singer 2015; Cullen et al. 2019; Tacoli et al. 2020). This is because most fungicides act by inhibiting respiration and or cell division, both of which are essential processes shared by all organisms (Sánchez-Bayo 2021).Nonetheless, the manner in which these non-target organisms respond to fungicide exposure exhibits variability that is contingent upon the specific species under consideration (Bostanian et al. 1998, 2009; Barbar et al. 2007). Results from this study support these assertions, given that we observed a difference in survival among the predators when they were exposed to different fungicides. These variations might be due to potential differences in the toxicokinetic and toxicodynamic mechanisms (Duso et al. 2020) within these predators which influences their selectivity to different compounds (Feyereisen et al. 2015; Van Leeuwen and Dermauw 2016). For instance, despite captan’s documented toxicity to bees (Mussen et al. 2004), our research indicates a differential impact of this fungicide in predatory mites where *N. cucumeris* had higher survival when exposed to captan compared to *N. californicus* and *A. swirskii*. The antifungal effect of captan is thought to arise from its interference with the sulfur part of glutathione, which then inhibits respiration in fungi (Roberts et al. 2007; Yang et al. 2011). Considering the importance of glutathione in animal cell respiration (Ribas et al. 2014), a similar action could explain its toxicity to some arthropods while perhaps other for example *N. cucumeris* might have evolved mechanisms to counteract this effect. Another notable example of selective toxicity was observed in the tetramethylthiuram disulfide treatment, where *A. swirskii* had higher survival compared to other predators. Although tetramethylthiuram disulfide is known to be toxic to animals, having been associated with cytotoxicity, angiogenesis inhibition, and dyschondroplasia (Zhang et al. 2018), research on its effects on predatory mites is still limited. However, in a study analogous to this one, mancozeb, a dithiocarbamate similar to tetramethylthiuram disulfide was found to be moderately toxic to the predatory mite *Typhlodromus pyri* Scheuten (Auger et al. 2004), an observation that aligns with our findings regarding moderate toxicity of tetramethylthiuram disulfide to *N. cucumeris* and *N. californicus*. Profoundly, our result suggest that application of tetramethylthiuram disulfide may not affect the survival of *A. swirskii*.

On the other hand, cyprodinil, an active ingredient of Switch (cyprodinil + fludioxonil) is an anilinopyrimidine fungicide that inhibits the biosynthesis of methionine in fungi (Waechter et al. 2010). Methionine is an essential amino acid whose absence interferes with proper growth and development (Martínez et al. 2017; Klein Geltink and Pearce 2019).This could potentially explain the observed decline in the survival of predatory mites following exposure to the cyprodinil + fludioxonil. Despite having the same active ingredient, there were differences in the survival of predatory mites exposed to captan (Captan 50 W) and captan® (Captan Gold® 4 L) treatments. This variation could probably be due to differences in their formulations. The adjuvants and solvents in captan® which are intended to enhance its stability, solubility, and efficacy, might have a negative impact on predatory mite survival compared to the formulation of Captan. All predatory mites showed decreased survival after exposure to hydrogen peroxide + peroxyacetic acid and cyflufenamid. Hydrogen peroxide has been found to have insecticidal properties (Caixeta et al. 2018) resulting from its induction of oxidative stress (Zhang et al. 2016). Additionally, the oxidative capabilities of hydrogen peroxide would avertedly allow it to interact with critical biomolecules such as nucleic acids, proteins, and lipids (Shi et al. 2022) in the cuticle of many arthropods, which could also explain the reduced predatory mite survival over time. Phenylacetamides represented by cyflufenamid have been demonstrated to inhibit DNA synthesis through the inhibition of thymidylate synthase (Ferreira et al. 2021). Despite the fact that residues of cyflufenamid have been reported to be safe for bees (Piechowicz et al. 2022) our results indicate that they negatively affect predatory mites.

Within the context of predator-prey interactions, many variables that adversely affect predation rates of predators can be associated with reduced predator population (Haque 2010).This was evident in our study, where the lowest predation occurred in fungicide treatments that had the most significant impact on predator survival. The highest predation was observed in the control treatment where *A. swirskii* and *N. californicus* consumed the highest number of *S. dorsalis* pupae. *Amblyseius swirskii* is a known predator of various thrips species(Wimmer et al. 2008; Dalir et al. 2021; Schoeller et al. 2022) and has been demonstrated to efficiently suppress *S. dorsalis* on strawberries in a greenhouse (Lahiri and Yambisa 2021). Similar to *A. swirskii*, *N. californicus* is a generalist predator that can survive feeding on thrips(Walzer et al. 2004; Rahmani et al. 2009; Azadeh et al. 2013) but exhibits preference for spider mites (McMurtry et al. 2013). With the exception of the cyprodinil + fludioxonil treatment, it was noted that the predation of *S. dorsalis* pupae by *N. californicus* increased over time, aliening with observations made by Rahmani et al. (2009) when *N. californicus* was provided with *Thrips tabaci*. However, in the context of this study, this shows that despite the reduced survival of *N. californicus* due to fungicide exposure, the predators that survived could still effectively feed on *S. dorsalis*. Another possible explanation for this observation could be that the strain of *N. californicus* used in the experiment was uniquely adapted to feeding on thrips given that previous research has shown that various strains of *N. californicus* exhibit distinct prey preferences (Castagnoli and Simoni 1999).This observation could perhaps suggest the existence of a commercially available strain of *N. californicus* that is uniquely adapted to feeding on thrips. Alternatively, the enhanced predatory efficiency of *N. californicus* could be due to early exposure to *S. dorsalis*. Although all predators were initially provided with *S. dorsalis* larvae as a food source during their development, *N. californicus* may have quickly adapted to feeding on *S. dorsalis* compared to other predators(Seiter and Schausberger 2015) given that early exposure can significantly enhance its predatory capabilities (Zhu et al. 2022).

Although *N. cucumeris* has also been successfully used to manage various thrips species (Messelink et al. 2006; Zilahi-Balogh et al. 2007; Delisle et al. 2015; Kakkar et al. 2016) including *S. dorsalis* (Arthurs et al. 2009), results from this study indicated low predation of *S. dorsalis* by this predator. The decrease in prey consumption by the surviving *N. cucumeris* could be due to several factors, one being the quality of predators sourced from commercial suppliers. (Dicke et al. 1989; Castagnoli and Simoni 1999; Lopez and Smith 2016; Lemos et al. 2023), and questionable suitability of thrips as a nutritious food source for the predators (Eubanks and Denno 2000; Wimmer et al. 2008; Schmidt et al. 2012; Vangansbeke et al. 2023). However, it is crucial to acknowledge that fungicides could directly impact predation in a manner that has not yet been fully identified.

In many phytoseiid mites, oviposition is intrinsically linked to predation (Sabelis 1990) and the ability of the predator to digest the prey (Janssen and Sabelis 1992; Rijn et al. 2005). Therefore, any factor that limit predation such as exposure to fungicides could affect oviposition (Sánchez-Bayo 2021). In all treatments, the lowest oviposition was observed in the cyprodinil + fludioxonil treatment, likely due to reduced predator survival. However, in other treatments, oviposition patterns remained similar across all predatory mites, especially after 47 h of exposure. Additionally, the highest oviposition occurred in the control group, compared to all other treatments. This suggests that the tested fungicides might not directly affect oviposition but may impact other critical parameters such as survival and predation, which in turn directly influence oviposition. Nonetheless, further research is required to determine whether predatory mites might avoid ovipositing on surfaces with fungicides, which could indicate a potential direct impact of fungicides on oviposition through altered behaviors.

In conclusion, this study shows that fungicides used in strawberry production can impact the efficacy of the predatory mites commonly released to suppress *S. dorsalis* populations. Notably, our results indicate the incompatibility of cyprodinil + fludioxonil with all the three predatory mites. The observed variation in survival, feeding and oviposition of predatory mites in most treatments shows that designing fungicide rotation programs that have minimal impact on predatory mites requires a predator -specific approach that tailors the rotation program to the individual sensitivities of each predatory mite. In addition, this research highlights the need for further investigation into the optimal timing for releasing predators post-fungicide application. Implementing a strategic “time to release” program could minimize the predators’ exposure to residual fungicides, thereby potentially reducing their adverse effects. Furthermore, additional research is required to determine the effect of exposure to fungicides on the life table parameters of the predators.

## Data Availability

Data relevant to this research are available upon request, and inquiries can be directed to the primary investigator.
